# Responsible Leadership and the Reflective CEO: Resolving Stakeholder Conflict by Imagining What *Could* be done

**DOI:** 10.1007/s10551-021-04865-6

**Published:** 2021-06-23

**Authors:** Nicola M. Pless, Atri Sengupta, Melissa A. Wheeler, Thomas Maak

**Affiliations:** 1 Professor of Management, Chair in Positive Business, Director Centre for Business Ethics & Responsible Leadership, University of South Australia, UniSA Business, North Terrace, Elton Mayo Building, Adelaide, SA 5000 Australia; 2 Assistant Professor, Area Chair, OB & HR, Indian Institute of Management Sambalpur, Sambalpur, Odisha - 768019 India; 3 Senior Lecturer, Faculty of Business and Law, Swinburne University of Technology, John St, Hawthorn, VIC 3122 Australia; 4 Professor of Leadership, Director Centre for Workplace Leadership, University of Melbourne, 198 Berkeley Street, Melbourne, VIC 3010 Australia

**Keywords:** Sustainable Development Goals (SDGs), Grand societal challenges, Emerging country multinational, Moral imagination, Responsible leadership, Business ethics, Social justice, Corporate social responsibility, Sustainable development, Cross-sector collaboration, Indigenous communities

## Abstract

In light of grand societal challenges, most recently the global Covid-19 pandemic, there is a call for research on responsible leadership. While significant advances have been made in recent years towards a better understanding of the concept, a gap exists in the understanding of responsible leadership in emerging countries, specifically how leaders resolve prevalent moral dilemmas. Following Werhane (1999), we use moral imagination as an analytical approach to analyze a dilemmatic stakeholder conflict (between indigenous communities in rural India and an emerging market multinational enterprise headquartered in the same country) through the lense of different responsible leadership mindsets and in light of different ethical principles and moral background theories. Based on this analysis, we arrive at a tentative moral judgement, concluding that the instrumental approach is morally inferior and recommending the *integrative approach as the morally superior choice*. In the subsequent discussion—focussed on what “could” (instead of “should”) be done, we apply the integrative script and use moral imagination as a pathway for generating morally justifiable solutions. Through this analysis, we provide novel insights on how to apply an integrative responsible leadership approach to a stakeholder conflict situation, using the single case study to expand the responsible leadership discussion to emerging markets.

## Introduction

In light of pressing societal problems (e.g. geopolitical instability, failing states, climate change, pandemics, social inequality) and growing power of multinational corporations (MNC) business leaders are increasingly asked to show responsible leadership – to do better – and to do more by contributing to solutions that benefit all stakeholders, through collective value creation (Donaldson & Walsh, 2015). Indeed, calls are made for responsible business leadership by scholars (e.g. Doh & Stumpf, 2005) and practitioners (e.g. Schwab, 2017) alike.(1) In the aftermath of the 2017 World Economic Forum in Davos, which was dedicated to the topic of “Responsive and Responsible Leadership”, the CEO of BlackRock (the world’s largest wealth management firm), argued that “profits and purpose are inextribably linked” (Fink, 2017) and urged his fellow CEOs to show responsible leadership for the benefit of all stakeholders. However, this is not an easy endeavour, particularly if stakeholder conflicts emerge in complex settings.

Responsible leadership (RL) can be understood as “a relational [and purpose-driven] influence process between leaders and stakeholders geared towards the establishment of accountability in matters pertaining to organizational value creation” (Maak et al., 2016, p. 464). While there is growing agreement among business leaders that responsibility at the individual and organizational level is important, there is a general knowing-doing gap in regard to responsible leadership (McKinsey, 2006, 2010), and only limited ‘orienting knowledge’ for executives to lead responsibly in emerging countries, let alone navigating complex or dilemmatic situations. The focus of this paper is a real-life case of a new CEO of an Indian multinational aluminum production company, who was exposed to such a dilemmatic stakeholder conflict in his home country. Instead of prescribing a certain moral approach (i.e. “should do”), our aim is to provide a discussion of perspectives suitable for practitioners, scholars and students alike to guide reflection on responsible decision-making by exploring “what could be done” in navigating dilemmas, crises and conflict situations, and subsequently developing morally imaginative solutions.

While corporate social responsibility (CSR) and RL are mostly discussed in light of MNC from developed countries (Egri & Ralston, 2008; Preuss et al., 2016), they are equally (if not more) important for top executives in non-Western and emerging country multinationals (Berger et al., 2011; Stahl et al., 2016). This is due to the rapid growth of emerging country MNC and their expanding role in the global economy (Gammeltoft et al., 2010; Miska et al., 2016); and the context in which they operate, which is often characterized by poor institutional conditions, weak rule of law, political instability and corruption (Cuervo-Cazurra et al., 2018; Marano et al., 2017; Stahl et al., 2018). The upper echelon of MNC, through their value systems and decisions, impact the broader ecosystem, locally and globally, and they play a pivotal role in resolving grand societal challenges (George et al., 2016). Hence, the call for responsible business leadership is based on the hope that businesses and their leaders can contribute to positive and sustainable change for the better.

However, calls for RL do not necessarily and automatically lead to positive development for people at the local level (e.g. Hennchen, 2015; Murphy & Vives, 2013). In poor countries with weak institutional contexts, business leaders may be inclined to simply adjust to the local context or “mirror” the low standards in their home countries (Preuss et al., 2016). This approach may be exarcerbated in crisis situations and is particularly pertinent to stakeholder conflicts.

Literature on responsible leadership in emerging markets is rare and has mainly focused on leaders of Western MNC conducting business in emerging economies (Moody-Stuart, 2014; Stahl et al., 2016) with only few empirical studies dedicated to responsible leaders doing business in their (home) emerging countries (e.g. Doh et al., 2011; Maak & Stoetter, 2012; Pless & Appel, 2012; Van de Loo, 2006).(2) These studies are predominantly about founders of responsible business organizations, or social enterprises, who have shaped the organizations through their mindsets and their moral values, virtues and principled behaviour. Little attention has been paid to RL in adverse contexts and situations (Coldwell et al., 2012; Varma, 2020). In this paper, we address this void by analyzing an aluminium production company’s decade-long struggle to commission a greenfield project in Odisha, India, and the dilemma faced by their new CEO.

The scenario was as follows: In the early 1990s, a MNC, specialising in metal refinery, acquired land and received approval from the Indian government to commence a greenfield project in rural India. However, the MNC faced criticism, resistance, and protests from both, NGOs and from the indigenous people who resided in the affected area, leading to massive delays and a decade-long conflict, including the death of three indigenous people following a protest that got out of control. A new CEO was appointed in 2000 to resolve the conflict and to drive the project ahead. Such stakeholder conflicts are steadily increasing worldwide (Banerjee, 2018), highlighting the challenges that leaders face and the importance of engaging in responsible leadership to overcome them. In the current paper, we aim to use this case study of actual events, described in more detail below, to analyze and reflect on the actions of the CEO and to recommend a dilemma resolution technique to enhance responsible leadership through the use of moral imagination.

Data for the case were gathered based on multiple rounds of data collection conducted during 2009 and 2012. We used both retrospective and observational methods. The retrospective data were collected through interviews after the incidents took place; and also included the company’s archival documents, newspaper clippings, internet information about the company and census data. Observations were made by one of the authors in person through discussions with multiple stakeholders while the incidents happened. Notes were taken to document the observations. More information on the research methodology is provided in the Appendix.

We use this case as a *heuristic for ethical analysis*. Instead of applying an empirical approach and thus using the case to generate new theory from inductive reasoning, we observe and analyze patterns and regularities in the case to derive theoretical conclusions (Alamgir & Cairns, 2015) and generate morally imaginative solution approaches.

Based on this single case study, we discuss how responsible executives of emerging country MNC in weak institutional contexts *could* approach crisis situations involving ethical dilemmas in order to achieve the best results for all legitimate stakeholders involved. More specifically, we use the case a basis for reflection on the decision dilemma in order to generate creative ethical solutions.

More specifically, we argue that leaders who intend to develop responsible solutions need to engage in moral imagination (Werhane, 1999). Moral imagination (MI) is a morally creative way of approaching wicked problems or dilemmas that may not have a clear solution – one that is morally right and an alternative option that is clearly morally wrong. Complex stakeholder settings may cause dilemmas which are dynamic and messy, preventing ‘quick fixes’ and clear solutions. Often these dilemmas are grounded in values – not right vs wrong, but right vs right (Kidder, 1995) – and require individuals to choose from “a diversity of goods” (Johnson, 1993). In other words, they are confronted with competing moral imperatives (Zhang et al., 2018). In these scenarios, moral imagination allows individuals to gain some distance and perspective and to come up with a "…third way—a kind of middle ground through the extremes…" (Kidder, 1995, p.167).

Moral imagination enables people to step back from dilemmatic presentation of two competing options (often based on deontological versus utilitarian thinking) and to ask themselves “what could be done?” (Zhang et al., 2018), thereby allowing the emergence of other possibilities beyond existing opposites. This is important since existing opposites can lend themselves to false dichotomies which often end in analysis paralysis (Bazerman & Moore, 2012).

We contend that this is particularly important for leaders of MNC who operate in organizations that are exposed to different legislations and diverse normative contexts – what is right at home, may not be right from a global perspective, particularly when human rights or SDGs are adversely affected. Hence, conflicts and dilemmas may arise from a clash of global norms and values but they may equally be the result of a clash in local norms and local values, or indeed, a combination thereof (Donaldson, 1996).

The purpose of this article is therefore (1) to investigate the link between RL and moral imagination in the pursuit of creative ethical solutions to dilemmas and grand societal challenges, and (2) to generate “orienting knowledge” (Mittelstraβ, 1982) on how leaders in emerging and developing countries *could* approach stakeholder conflicts, dilemma and crisis situations in a morally imaginative way. Moreover, we provide insights on how to apply a responsible leadership framework when responding to local CSR standards while adhering to global CSR norms (e.g. Donaldson, 1996; Stahl et al., 2018).

With this article, we contribute to the literature on responsible leadership by systematically analyzing the ethical bases of different RL mindsets in face of a crisis situation, introducing moral imagination to the RL discourse, and further developing the understanding of an integrative RL logic. Secondly, we contribute to the leadership discussion in the field of international management by explicitly addressing and reflecting on the moral challenges and tensions between local CSR responsiveness and global CSR standardization that executives of emerging MNC can face in crisis situations in their home countries, and providing “orienting knowledge” for tackling such situations based on an integrative and morally imaginative RL approach.

This article is structured as follows. We provide a brief literature review on responsible leadership (and its theoretical foundation) and moral imagination. We then present the case, and subsequently conduct an ethical analysis from two contrasting (but not opposing) responsible leadership viewpoints: instrumental and integrative responsible leadership. The analysis is guided by two concepts: normative business intentions and central ethical philosophies. We then discuss consequences of the responsible leadership approaches for strategic decision-making and stakeholder relations in a crisis situation and propose ethically reflective and morally imaginative ways in which an integrative leader *could* respond to the situation. We conclude with theoretical and practical implications.

## Responsible Leadership

Traditional leadership research focuses mainly on the individual level and examines the relationship between leaders and direct reports (followers) and how leaders exert dyadic influence over them “to guide, structure, and facilitate activities and relationships” to achieve certain objectives (Yukl, 2012, p. 6). RL broadens the leader–follower relationship and encompasses a broader group of stakeholders as followers (e.g. Doh & Quigley, 2014; Freeman & Auster, 2011; Maak & Pless, 2006; Voegtlin, 2011; Waldman & Galvin, 2008) that leaders interact with, have responsibility for, and try to mobilize (Pless & Maak, 2011). This approach acknowledges that leadership projects unfold within a broader stakeholder environment in which business leaders operate. Moreover, it acknowledges the complex relational nature of leadership and that leadership responsibilities extend beyond the dyadic relationship of leader and follower – and that as a consequence leadership motives and values may be contested. Behaving responsibly means not only avoiding harm, but also doing good and being good – displaying a virtuous character (Cameron, 2011). In addition, leaders must be prepared to mirror relational complexity through behavioral complexity. Behavioral roles associated with RL, and introduced in the roles model of responsible leadership (Maak & Pless, 2006), include *normative* roles (citizen, steward, visionary), *relational* roles (servant, weaver/boundary spanner) and *operational* roles (change agent, architect and coach). These roles are overlapping, they form an integrated whole – a “gestalt”. Depending on time and place the leader enacts different roles or different sets of roles as required (Maak & Pless, 2006).

A key idea of RL is that leaders influence and mobilize stakeholders inside and outside the organization to achieve results for business and society. The call for leaders to embrace CSR (Fink, 2017) and act as corporate citizens (Schwab, 2017) increases the set of objectives that leaders pursue (e.g., contributing to the SDGs), broadens the stakeholder focus and (depending on leaders’ approaches and objectives) widens the sphere of influence. New challenges emerge for leaders of MNC, such as leading a business responsibly in a multi-stakeholder context, pursuing multiple objectives (e.g. financial profit and social purpose), ensuring sustainability for the firm and society, and decision-making in light of adverse impact on stakeholders and the environment.

In essence, upper echelons are challenged in regard to leadership issues emerging at the strategic level and pertaining to questions of accountability, values and purpose of the firm (Freeman et al., 2007), which in turn influence resource allocation in terms of, e.g., money, time, attention to, and engagement with stakeholder groups. As a consequence, effective and responsible approaches are required and essential in situations of conflicting stakeholder interests and demands where leaders need to mitigate tension and reconcile conflicts and dilemmas.

### The Role(s) of a Responsible Leader

Responsible leadership roles that are particularly relevant to the above mentioned leadership challenges are the *normative* roles of the leader as steward, citizen, and visionary. The leader as *steward* is a custodian of values and resources with a strong ethical decision-making compass (Paine, 2006). The enactment of this role implies to protect what one is entrusted with (this can range from organizational values and heritage to environmental protection) and to ensure that there is consistency between the philosophy of the firm (including values and purpose) and the actions and deeds of organizational members in interaction with stakeholders (Maak & Pless, 2006). The leader as *visionary* has foresight and a long-term perspective. Role behaviour include the motivation and inspiration of followers through a clear sense of purpose directed towards all legitimate stakeholders of an organization. Lastly, the leader as *citizen* recognizes that business is part of society and has a co-responsibility in addressing and resolving societal problems. As part of this role he/she demonstrates caring behaviour aimed at the well-being of local and global communities that are impacted by business operations (Pless, 2007).

Other roles have a particular *relational* character, such as the roles of servant, weaver/boundary spanner and communicator, and are of particular relevance for stakeholder interaction and engagement. The *servant* leader cares about the needs and interests of internal and external followers and shows a high degree of relational intelligence (Pless & Maak, 2005) when interacting with different stakeholders. The leader as *weaver* takes on boundary spanning roles at the interface of firm and external stakeholders. Leaders at the upper echelon navigate dynamic webs of relations around their organization, including other firms, the environment, and communities in which they operate (Finkelstein et al., 2009; Maak et al., 2016). They are responsible, both for facilitating the relational processes with and among stakeholders and for the quality of these relationships. As *communicators* leaders articulate the particular purpose and vision of the firm, provide direction to followers and interact with stakeholders in business and society. It is also through communication that conflicts of interest among stakeholders can be mitigated (Maak et al., 2016).

Moreover, and in more *operational* terms, the strategic direction or redirection of the firm may require that leaders act as *change agents*. However, in contrast to transformational leadership theory (e.g. Bass, 1990), initiating change is not seen as an end in itself but as a means to build and cultivate responsible business (Maak & Pless, 2006). There is hope that businesses and their leaders through their power, resources and influence can contribute to positive and sustainable change for the better. As *architect* the leader ensures that a moral infrastructure is in place and that systems and processes are fair and inclusive. And in the role as *coach* the leader supports followers in achieving their individual and organizational objectives, and in engaging with the community. In comparison to the other roles in the model, the role of the architect and coach are not immediately relevant for the handling of the crisis situation (though they become relevant in terms of organizational development), and therefore not discussed in such depth as other roles.

### Responsible Leadership and Stakeholder Theory

There is agreement that responsible leadership is grounded in stakeholder theory (e.g. Doh & Quigley, 2014; Freeman & Auster, 2011; Maak & Pless, 2006; Voegtlin, 2011; Waldman & Galvin, 2008) – a view that is echoed by recent statements of senior business leaders, as indicated above. More specifically, our approach to responsible leadership is based on normative stakeholder theory and reflects on underlying moral or philosophical principles (Donaldson & Preston, 1995, p. 72). As such it is particularly relevant for RL pertaining to decisions on value judgements, legitimacy and stakeholder preferences at the level of the upper echelons (e.g. Maak et al., 2016; Waldman et al., 2006). Leaders’ values and normative mindsets (including intentions and attitudes) drive their behaviour (e.g. decision-making), which in turn impact organizational approaches towards stakeholders and CSR (e.g. Chin et al., 2013; Sully de Luque et al., 2008) and are especially relevant in situations that require decisions under time constraints, as in the crisis situations exemplified by the following case.

Maak et al. (2016) have identified two normative responsible leadership approaches, an “instrumental” one and an “integrative” one. Leaders with an instrumental RL approach are described as being focused on organizational objectives (e.g., maximizing profits, realizing growth) and business performance, paying little or no attention to non-core business issues. They feel only accountable to shareholders of the firm (fiduciary duty towards the owners of the firm) and have a limited range of stakeholder interactions, mainly focused on shareholders/owners of the firm and a selected group of core business constituents, such as employees, suppliers, governments. They understand these constituents as means to an end to achieve business ojectives and interact in an instrumental, transactional and/or rule-based manner (Pless et al., 2012). In contrast, leaders with an integrative RL approach are described as leading with a broader focus on value creation and integrating business and societal objectives. They feel accountable to all legitimate stakeholders (social welfare orientation) and engage with a broad range of constituencies, including fringe stakeholders. They understand these constituents as ends in themselves, engage in active communication and collaboration with stakeholders, and pursue a collaborative and inclusive approach.

### Central Ethical Orientations in Responsible Leadership

Moral and ethical concepts of leadership are criticized for either reflecting a Western-based perspective (e.g., Young, 2006) or being conceptually vague without articulating specific norms that moral leaders can refer to (e.g., Giessner & van Quaquebeke, 2010). As a response, and to avoid ethical relativism (Donaldson, 1996), Eisenbeiss (2012) derived a set of four principles (humane orientation, justice orientation, responsibility and sustainability orientation and moderation orientation) called central ethical orientations of leadership. The review comprised ancient Western traditions (Plato, Aristotle) and Eastern traditions (Confucianism), as well as modern Western traditions (Kant, Rawls, Jonas) and modern Eastern traditions (Tagore). Western world religions that were studied comprised Christianity, Judaism and Islam, and Eastern world religions comprised Buddhism, Hinduism and Sikhism. These central ethical orientations reflect an intercultural and interdisciplinary view of the normative foundations of moral leadership concepts and “present the cross-disciplinary and cross-cultural ‘lowest common denominator’” (Eisenbeiss, 2012, p. 794), or the minimum standard to which most cultures converge. In our study, they serve as normative reference points. The intersection of these orientations with literature on RL is discussed in the following.

#### Humane Orientation

A humane orientation is a virtuous and relational approach based on the understanding that others should be treated with respect and dignity (Melé, 2016; Pirson, 2017; Pless et al., 2017a, 2017b), and seen as ends, not as means to an end (Eisenbeiss, 2012; Jones et al., 2007). A humane orientation can be observed by responsible leaders’ way of interacting with stakeholders – their *compassion and true concern and care for the well-being of others* and the recognition and protection of their rights. As such it transcends self-interest, includes an altruistic spirit (Melé, 2009), as well as respect for dignity and human rights (Eisenbeiss, 2012; Honneth, 1997), and identifies its roots philosophically in Kant’s (1977) categorical imperative, the Confucian golden rule (Ivanhoe & Van Norden, 2001), and also in religions such as Christianity, Buddhism and Sikhism. Humane orientation is also a main dimension in the intercultural leadership study GLOBE (House et al., 2004).

#### Justice Orientation

A justice orientation is a form of fair and consistent decision-making and treatment of others. For leaders this means *treating people equally and refraining from discrimination* (De Hoogh and Den Hartog 2009; Eisenbeiss, 2012; Olsaretti, 2018). The concept of justice has a rich tradition in Western religion and moral philosophy. However, Eisenbeiss (2012) points out that it also plays a central role in other religions like Islam and Sikhism. Fairness and justice are recurring topics in research on global leadership (e.g., Stahl et al., 2016), leadership ethics (Ciulla, 2006; Ciulla et al., 2018), theories of ethical leadership (Brown et al., 2005; De Hoog and Den Hartog 2009; Simola et al., 2010), and responsible leadership in evaluating consequences of utilitarian approaches and equity arguments focused on stakeholder inclusion and fairness (e.g. Waldman & Galvin, 2008, pp. 330ff).

#### Responsibility and Sustainability Orientation

This dimension refers to the concern of leaders for society and the environment, which guides responsible conduct (Eisenbeiss, 2012). In RL research, this orientation has been conceptualized as *accountability to others, including to future generations, the welfare of society, and the environment* (Pless et al., 2012). Eisenbeiss (2012) shows that this orientation has roots in different philosophical and religious traditions, including Hinduism, Buddhism and Sikhism. The Western philosopher Hans Jonas (1984) makes a time-related distinction that is relevant to this orientation. He distinguishes between a formal *ex-post* responsibility and a substantive *ex-ante* responsibility. Ex-post responsibility means to take responsibility for past wrongdoing (e.g., in the form of a legal punishment, such as payment of a fine). In contrast, substantive responsibility is proactive and care-driven. It implies that leaders consider *ex-ante* the impact of their decisions on others, including future generations. This form of *ex-ante* responsibility is reflected in the Brundtland Report as “development that meets the needs of the present without compromising the ability of future generations to meet their needs” (WCED 1987, p. 8).

#### Moderation Orientation

Moderation is one of the key concepts in RL research. It refers to the *humility, decency and temperance of a leader* and to balanced behavior (Eisenbeiss et al., 2014; Rego et al., 2012). At the micro level, it is expressed in leaders’ self-control and their ability to contain emotions, control self-interest and balance motivations, as well as the interests and demands of others (Cameron, 2011). At the macro level, it becomes visible in leaders’ attempts to balance opposing interests at the organizational and societal levels, often occurring in stakeholder interaction and the need to reconcile conflicts of interests, dilemmas and paradoxes (Schraa-Liu & Trompenaars, 2006; Wettstein et al., 2019). According to Eisenbeiss (2012), moderation and balance are important in ancient Western philosophy, Buddhism, Confucianism and Sikhism. Humility and moderation are discussed as essential virtues of responsible leaders that are necessary to balance the inner self with the needs of various stakeholders and to reconcile conflicts of interests (Cameron, 2011; Rego et al., 2012).

### Moral imagination

The first use of the term moral imagination is associated with the book Reflections on the Revolution in France by the Irish statesman and philosopher Edmund Burke (1727–1797) (Stephenson, 2007). While evidence combining both leadership and imagination is scarce, there is a growing interest in disciplines such as philosophy, psychology, and management in the topic of moral imagination (e.g., Bevan, Wolfe, and Werhane 2019; Caldwell & Moberg, 2007; Johnson, 1993; Kidder, 1995; Lederach, 2005; Moberg & Seabright, 2000; Werhane, 1998, 1999, 2002; Yang, 2013) and moral insight (Zhang et al., 2018). Different authors (e.g. Eisenbeiss et al., 2014; Schraa-Liu & Trompenaars, 2006; Stephenson, 2007) stress the importance of ethical abilities and particularly moral imagination for leading responsibly in an environment of conflicting values.

#### Moral Imagination and Decision Making

Werhane (1999) defines moral imagination as ‘‘a necessary ingredient in responsible moral judgment’’ that can enable in particular circumstances to ‘‘discover and evaluate possibilities not merely determined by that circumstance, or limited by its operative mental models, or merely framed by a set of rules or rule-governed concerns. In managerial decision-making, moral imagination entails perceiving norms, social roles, and relationships entwined in any situation. Moral imagination allows individuals to create alternative solutions when faced with a moral dilemma, which often presents two competing options that one needs to choose from. Some moral dilemmas we encounter have one clear option that is morally right and an alternative option that is clearly morally wrong. In these instances, it is not difficult for individuals to perceive what ought to be done; that is, the right vs. wrong distinction is not ambiguous or cloudy. What complicates the picture is when the two options of the moral dilemma are both grounded in values – not right vs. wrong, but *right vs. right* (Kidder, 1995). Faced with these dilemmas, individuals must choose from “a diversity of goods” (Johnson, 1993); in other words, they are confronted with competing moral imperatives (Zhang et al., 2018). In these scenarios, moral imagination allows individuals to gain some distance and perspective and to come up with a "…third way—a kind of middle ground through the extremes…" (Kidder, 1995, p.167).

Zhang et al. (2018) argue that a shift from a “should” to a “could” mindset allows people to step back from dilemmatic presentation of two competing options (often based on deontological versus utilitarian ethical frameworks) and to ask themselves not “what should be done?” but instead “what could be done?”, thereby allowing the emergence of other possibilities beyond existing opposites. The use of moral imagination or moral insights opens up other solutions that do not force an individual to violate their moral principles or to make a trade-off between two imperfect options. We suggest that the use of moral imagination can help leaders to make better decisions in difficult situations as outlined in the case scenario.

#### Moral Imagination and Responsible Leadership

Moral imagination requires particular capacities relevant for leadership and approaching moral dilemmas. First, a *mental state of heightened awareness* (Werhane, 1999, p. 93) or mindfulness (Eisenbeiss et al., 2014; Pless et al., 2017a, 2017b): “(1) awareness of the character context, situation, event, and dilemma at issue; (2) awareness of the script or schema function in that context and role relationships entailed in that context, and (3) awareness of possible moral conflicts or dilemmas that might arise in that situation, including dilemmas created at least in part by the dominating script or the situation itself.” (ibid, p. 103). Second, a *capacity for productive imagination*. This means to overcome the tendency to generalize a dominant operating script or perspective and to be able to challenge one’s perspective on an issue, activity or decision; and be aware other mental models. Third, an *ability for creativity* as envisioning and actualizing “novel, morally justifiable possibilities through a fresh point of view or conceptual scheme” (ibid, p. 105).

These capacities are of particular importance for leading responsibly in a stakeholder environment of contested values, “as in such circumstances there might not be *the* one right thing to do [when faced with a dilemma situation entailing conflicting stakeholder interests], but leaders may in fact need to overcome [such] conflicts […] by employing moral imagination […] on order to generate new, non-linear but still ethically sound solutions.” (Pless & Maak, 2008, p. 21).

## Case Narrative: The Case of Hindalco Industries’ UAIL Refinery and its new CEO

### The Company, Context and Conflict

We will now present a more detailed account of the case of Hindalco Industries and their UAIL refinery in rural India and the dilemma faced by the new UAIL CEO in dealing with the decade-long struggle to commission a greenfield project in Odisha.

#### Hindalco Industries, Aditya Birla Group (ABG)

Hindalco Industries Ltd is a US $15 billion metal flagship company of ABG founded in 1998. In 2017, it was ranked 14 in Fortune India 500 and has a presence in 10 countries outside India. Its product portfolio includes aluminium, copper, cargo handling, acids and fertilizers. It is the world’s largest company in aluminium rolling. It deals with bauxite mining, alumina refining, coal mining, captive power plants and aluminium smelting to downstream rolling, extrusions and foils. The company’s mission is to pursue the creation of superior shareholder value while being a responsible corporate citizen (Hindalco, 2020). The company has received several national and international awards for its initiatives to promote community welfare, environment protection, quality and export performance. With an ambition of becoming a premium global metal producer, Hindalco began an ambitious expansion of its aluminium metal business with multiple major greenfield projects, including the UAIL refinery, which was planned in the Kashipur Block region of Odisha.

#### The Socio-cultural Context of Odisha

**Figs. 1 and 2:**
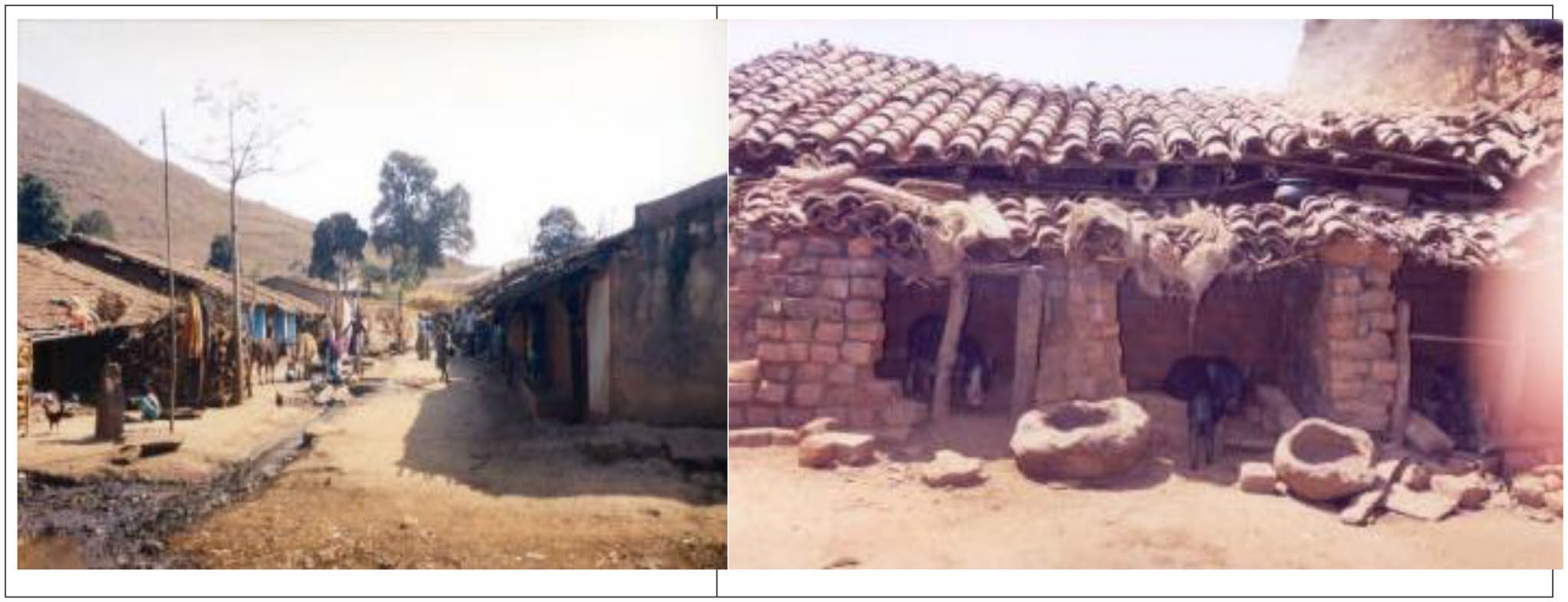
A village in Kashipur Block. Source: UAIL (Mangaraj et al., 2009; photographs used with permission from UAIL)

Odisha is one of the poorest states of India with 33% of the population living below the poverty line (see Figs. 1, 2) and only 20% of people owning land (World Bank, 2016). Unemployment is high, and 61.8% of those who have work are involved in agricultural activities; literacy is very low (72.87% in the 2011 Census of India: see Census, 2011). The state population has a high proportion of Scheduled Tribes (also known as indigenous or ‘tribal’ people or Adivasi, 22.1%) and Dalit or Scheduled Castes (16.5%, as per the 2001 census: see Census, 2001). On the other hand, the state is rich in mineral resources and fertile land. However, it lacks the resources and technology to explore the deposits (Kaushal, 2017). To develop the state and its economy, the government has invited domestic and foreign investors and encouraged power, steel and aluminium companies to set up plants and factories (Kaushal, 2017). Public policy and implementation in regard to environmental and human rights issues are still in a developmental stage. Wages as well as legal and environmental standards are low in Odisha, which attracts interest from global business.

#### Bauxite Mining in Odisha: The Utkal Alumina International Limited (UAIL) project

UAIL, a greenfield aluminium refinery, co-generation power plant and bauxite mine, began in 1992–93 in the most remote and hilly terrain of Odisha. After receiving approval from the state government, and acquiring land for the plant, construction of the long-distance conveyor system, railway siding, raw water intake and waste disposal areas began in 1993 under the supervision of the state government. A total of around 3000 acres of land spread over 24 villages in Kashipur Block of Odisha was selected for the purpose. However, since then, the project has repeatedly faced stiff resistance, criticism and opposition from NGOs and anti-industrialization and anti-capitalist groups sponsored by agencies from different parts of the country, leading to massive delays.

#### The Conflict

**Fig. 3:**
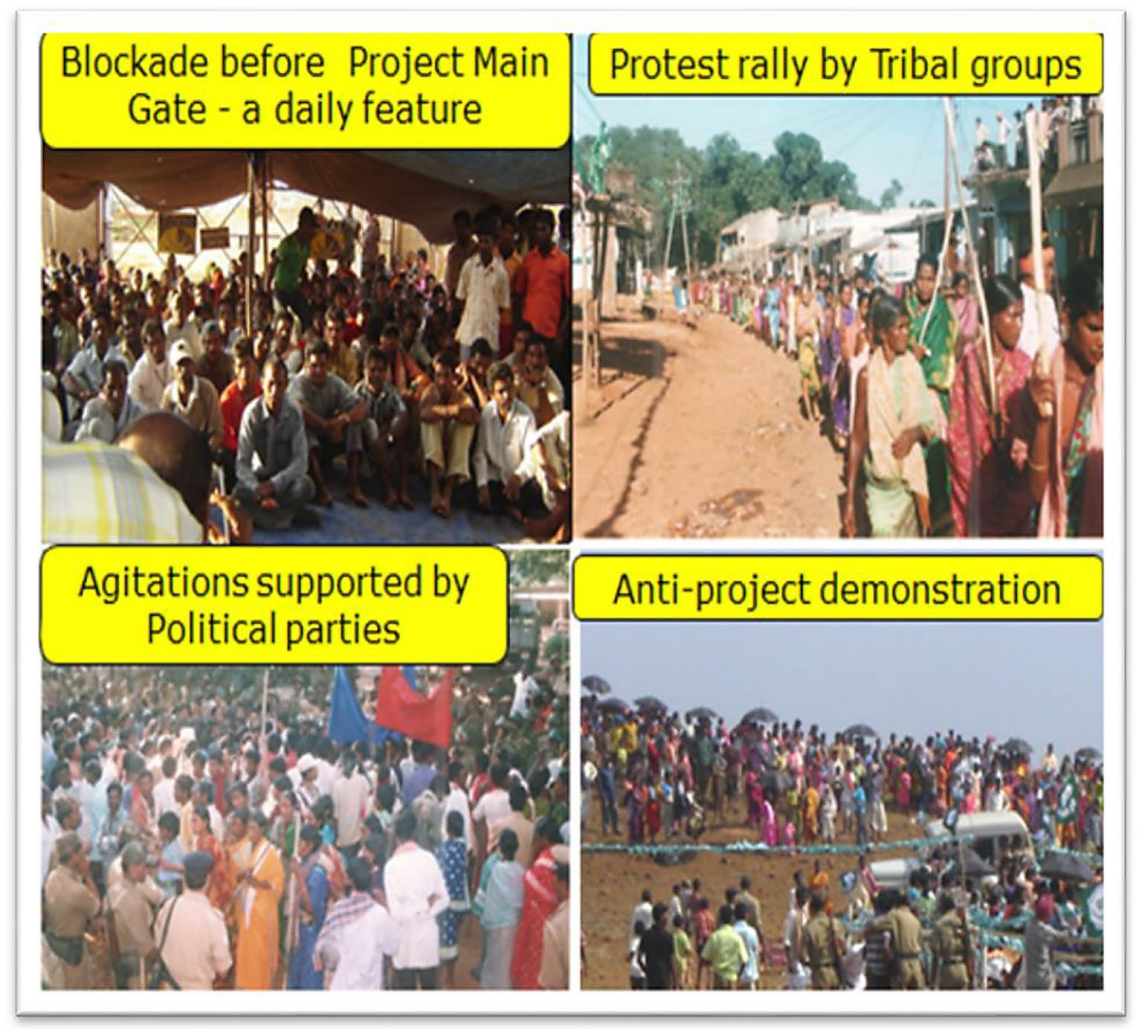
Conflict in Odisha in Kashipur Block of Rayagada district. Source: Hindalco photo archive (photographs used and adapted with permission from UAIL)

UAIL faced controversy from its inception when land was acquired under the direct supervision of the government in 1993. The population affected by the project was largely comprised indigenous people and Scheduled Caste communities (fringe stakeholders). Even after completion of land acquisition and acquiring the statutory and regulatory clearances, project construction could not begin due to resistance from villagers affected by the project and regular protests (see Fig. 3). The villagers were supported by NGOs and social activists who were opposed to industrialization in tribal areas. The protests addressed diverse issues, namely, sustainable development, expected negative consequences, expropriation of tribal lands, and the ecological and socio-economic impact of the industrial venture. The situation deteriorated further in December 2000 when a protest got out of control and police fired at the protestors, resulting in the deaths of three tribal people. As a result, the company could not start plant construction and community members’ trust in the company and local administration further deteriorated.

#### The New Leader

In light of extensive project delays and conflict escalation, the Chairman of ABG was in search of an effective leader who could resolve the situation and drive the UAIL project ahead. A new CEO was hired who had both a local background and substantial industry experience. He (3) had gained insight into most of the relevant aspects of running the project by working for over 40 years in the pulp and paper industry, including implementation of greenfield projects. After six weeks in the job and extensive information gathering, he identified the following challenges:

*Operations challenge*. Lack of infrastructure (e.g., medical facilities, schools) and the conflict created difficulties for managers and contractors to work. Employees reported:

On a few occasions, we were under severe pressure to stop work due to local protests. We were helpless, as the villagers had difficulty understanding either our language or the importance of maintaining progress on the project. Many of our officers were threatened, and some of us were beaten by the villagers, who demanded that work cease.

Contractors were also affected. They reported that during work engineers were physically abused for money which led to frequent work stoppages. Also, theft of materials and equipment led to project delay and rise of costs. Not surprisingly, attracting and retaining managers and contractors became a true challenge.

*The environmental sustainability challenge.* Aluminium is one of the crucial metals for today’s competitive world, and demand is high – the reason why the company engages in these projects. However, it also bears societal risks such as land alienation, destruction of local cultural heritage, as well as substantial environmental risks, such as deforestation, energy consumption, production of alarming levels of greenhouse gases (Norgate et al., 2007), generation of toxic waste, and air, water and soil pollution. The latter also pose health and safety risks for the local population whose livelihood depends on their land, on agriculture and fishing; villagers even get their drinking water from lakes and rivers. Industrial production close to their habitats can have a direct and detrimental impact on their health and safety. One of the environmental activists protesting against the set up of the aluminium plant in the location commented:

This plant will pollute the environment with green[house] gases; they will not only use the river water for the production, but will also throw toxic waste to the river …; land of the farmers are given to them … who is going to be benefitted out of this?

Indeed, the CEO thought, in light of India’s commitment to becoming a low-carbon economy, a responsible course of action is needed to create long-term sustainability.

*The trust challenge.* The company’s perspective was that, given the extreme poverty and locals’ struggle to survive, the UAIL project could foster regional development and provision of jobs, education and medical help through industrial engagement. However, villagers, NGOs (some of them funded by national and international agencies), anti-capitalist/anti-industrialization groups and other social agencies had a different view, fearing negative impact of the UAIL project on health, environment and livelihoods, and requested that the land be given back to the original owners. A tribal villager explained his struggle and fears as follows:

Getting sufficient food twice a day for all the members of my family was difficult. Several days a month we could offer food to our children only and with nothing available for adults. The small area of land we had for cultivation could not generate enough earnings for our survival. … Now they are planning to take our land from us. We are sons of soil, what we will do if land is taken away from us?

The CEO realized that the crisis and conflict with indigenous stakeholders was a profound issue not only leading to massive delays and economic losses, but also to human hardship, unsatisfactory stakeholder relations and reputation damage, reaching a tipping point following an incident involving a police shooting. In essence, he was faced with a moral dilemma. On the one hand, he might order the project to be stopped to preserve the status quo of the environment and indigenous lifestyle (including poverty and lack of education) as anti-capitalist/anti-industrialization groups were requesting. The other option was to face the challenge and move forward with the industrialization project. However, the second option came with an environmental footprint. Yet, it also offered the chance to contribute to regional development and the potential to break the poverty cycle. He was wondering what he should do in light of this trade-off between two imperfect options.

### Philosophical Approach to the Analysis

In analyzing the data, we noticed the complexities of the stakeholder conflict, the difficulties for the leadership of imagining a comprehensive, ethical solution, and the lengthy struggle to determine and reconcile legitimate stakeholder interests – suggesting that the organization and its leadership may have been captured in a particular dominant mental model that did not allow for new ways of seeing. To approach this complexity we decided to utilize an “ethos of imagination” (Alamgir et al., 2019) to inspire and transform current practices. Therefore, we opted for a philosophical approach rather than a traditional empirical case-based analysis, and to apply an analysis of moral imagination.

Werhane (1999) argues that moral imagination is an essential condition of innovative decision-making of business executives. She considers the mediated nature of culture, ideology, and human experience, and particularly argues that how we perceive the world is influenced by conceptual frameworks and assumptions: “We all perceive, frame, and interact with the world through a conceptual scheme modified by a set of perspectives or mental models” (1999, p. 49). Responsible leadership mindsets reflect such mental models, which can help leaders on the one hand to focus their attention and energy, pursue a particular idea, vision, purpose, and create a specific script for their ‘leadership movie’. However, limiting the perspective to one view can result in myopia and constrain the moral outlook. Our approach of reflecting on the dilemma from different RL mindsets can help identify, understand and assess different perspectives and approaches for identifying the most sustainable one.

We use moral imagination as a philosophical approach to the case analysis. (Werhane, 1999). Moral judgments involve a delicate balance of context, evaluation, projection of moral standards, and imagination. The latter “involves heightened awareness of contextual moral dilemmas and their mental models, the ability to envision and evaluate new mental models that create new possibilities, and capability to reframe the dilemma and create new solutions in ways that are novel, economically viable, and morally justifiable” (p. 93); Werhane further states that “the linchpin of this process is a highly developed moral imagination that perceives the nuances of a situation, challenges the framework or scheme in which the event is embedded, and imagines how that situation and other similar situations might be different.” (1999, p. 126). Moral imagination enables individuals to compare and contrast own perceptions, experiences, mindsets, and cultures with that of others. It also increases the breadth of possibilities that one sees or generates, and ultimately provides more choices regarding potential courses of action (Arnold & Hartman, 2003). Our analysis of the options available to the CEO to address the conflict aims at transcending dominant mindsets and opening up for new ways of seeing.

Moral imagination is particularly suited to analyze complex and systemic cases in business ethics that not only involve the individual decision-making level, but also organizational and societal levels. To analyze this decision-making case and to reflect on managerial norms, social roles, and relationships entwined in the situation, we provide substantive background information on the case: describing its context (country and culture, living conditions, economic context, etc.), the conflict situation and the ethical issues involved (environmental degradation, impact on villagers, land acquisition). We also give an overview of the affected stakeholders, noting any human rights violations, and focus on the least privileged stakeholders – those most profoundly affected by the project, namely the indigenous villagers.

**Fig. 4:**
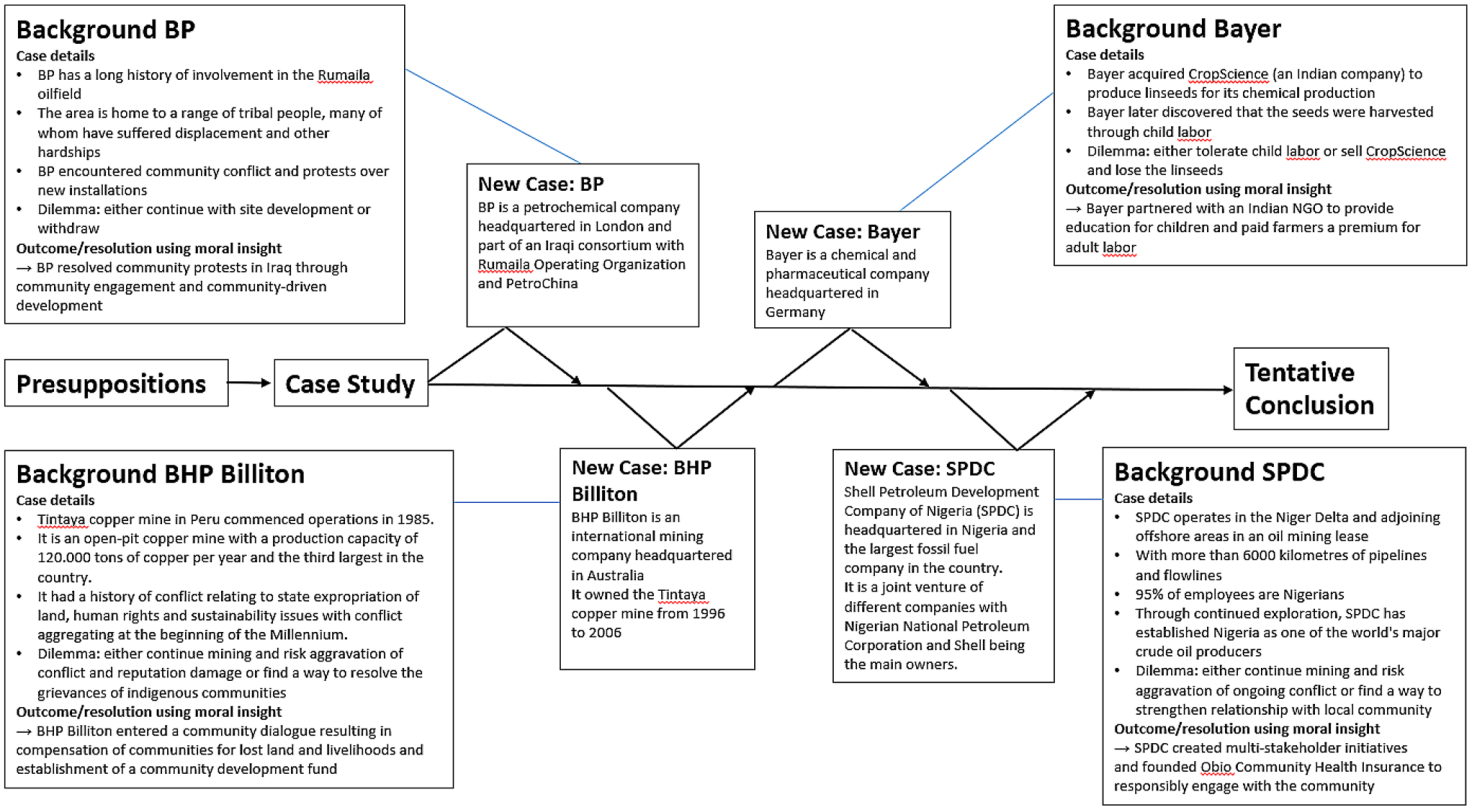
Background information on case vignettes used following an iterative case-based approach. Source: Adapted from Werhane (2017, p. 215)

By juxtaposing two RL approaches (instrumental and integrative), both rooted in different normative assumptions, we then take the perspective of a non-involved observer (with a “disengaged view”, Werhane, 1999, p. 122) in the case and analyze the consequences, weighing benefits and harms, of these RL approaches, asking what a person with each respective approach would decide. Following Werhane (1999) we start with the specific case, and use the RL orientations as a lense to reflect on the dilemma situation in light of different ethical principles and moral background theories, moral minimums, stakeholders and ethical issues involved in the case. Based on this analysis (see Sect. 4), we arrive at a tentative moral judgement and recommend that, in this case, the *integrative approach is the morally superior choice*. In the subsequent discussion, we apply the integrative script and use moral imagination as a pathway for generating morally justifiable solutions. The analytical process is enriched by an analysis of a series of case vignettes from comparable contexts in developing countries with high power distance (Hofstede, 2019), and organizations from high-impact industries, such as BHP Billiton, BP, Shell Petroleum Development Company of Nigeria Limited and Bayer (see Fig. 4), and the use of learnings from each case to hone the analysis (Werhane, 2017, p. 214). This analytical process helps to inform in practical terms what *could* be done, as opposed to the usual focus on what *should* be done (Zhang et al., 2018). As mentioned earlier, this approach allows us to step back from dilemmatic presentation of two competing options (i.e. what should be done) and to ask “what could be done?” This may open up other solutions that do not force one to violate one’s moral principles, or to make a trade-off between two imperfect options.

More specifically, the case dilemma presents itself for the CEO with the following two imperfect options (i.e. two competing moral imperatives): should he order a stop of the project as anti-capitalist/anti-industrialization groups were requesting or should he move forward with the industrialization project? Apart from the ideological side that these groups put forward, the project challenges were complex, and a quick and cost-effective outcome from this situation could not be promised to shareholders. On the other hand, if the company stopped the project and backed off, as some joint venture partners already had, it would mean accepting the financial loss of shareholder money that was already invested. Yet, it would also mean that the current life situation of the villagers could be maintained and the state of the natural environment preserved. However, the economic status quo of the region characterized by subsistence agriculture, devastating poverty, high unemployment, lack of education and high rates of illiteracy among the local population would also be cemented. At the same time, the stoppage of the UAIL project would not necessarily mean that the land that the villagers had sold would be given back to them, since it was acquired under supervision of the government. It would also mean that the villagers were not protected from future change and industrialization. Since the government is driving the industrialization of the region, it is likely that they will find other investors who continue the project—these could also be businesses from other parts of the world and those that do not pursue a responsible citizenship approach as the company does.

Rather than getting stuck in an ideological debate and prescribing what “should be done”, we want to reflect on different options that are available for the CEO to approach the situation: from a responsible leadership perspective and in light of different stakeholder interests. Thereby, we intend to provide “orienting knowledge” (Mittelstraβ, 1982) and use moral imagination—what could be done to continue the project, develop the region economically and potentially provide a pathway out of poverty.

## Philosophical Analysis of the Case Study

In the following we will analyse the case and outline possible approaches from the two RL logics (instrumental and integrative). Applying an ethical perspective, we will conduct a fine-grained analysis of these approaches by focusing on central ethical orientations as they guide leader decision-making.

### Instrumental Responsible Leadership: Central Ethical Orientations

#### Humane Orientation

According to the RL literature (e.g. Waldman & Galvin, 2008), the instrumental logic implies that leaders are driven by materialistic values (e.g., profit maximization) and self-interest. This approach is supported by traditional corporate governance systems rooted in a shareholder value model (e.g. Scherer & Voegtlin, 2018), ensuring that concern for others is mainly focused on stockholders and contracts with them are honoured (Jones et al., 2007). Leaders following an instrumental logic recognize other stakeholders as relevant if they are beneficial to the business and help create shareholder value or fulfil shareholder demands and/or if the situation requires this (e.g., urgent stakeholders in Agle et al.’s (1999) model). In the case, the instrumental approach would involve caring mainly for the needs of the business and interests of the project financiers to ensure that the project can be driven forward in a more efficient and effective way. The interests of fringe (but legitimate) stakeholders like the indigenous villagers are likely to be subjugated to the interests of shareholders. Conflict resolution would be sought only to get the project going, achieve business objectives, achieve business success and satisfy financiers.

#### Justice Orientation

The mindset of the instrumental leader is rooted in a utilitarian philosophy (Rosen, 2003), which involves the promotion of overall human welfare through maximizing benefits such as profits. Actors’ behavior is guided by an economic cost–benefit calculus and aimed at “the greatest total beneficial consequences minus harmful consequences” (Jones et al., 2007, p. 138). Instrumentally guided leaders follow rigid utilitarian rules reflecting market efficiency that guide behavior and decision-making.

In the case, an instrumental approach had been applied in the past. The project was set up based on the assumption that it would lead to the greatest total beneficial consequences for the state. An export strategy promising high profits due to high demand for aluminium in the world market, low costs for land acquisition and wages, and the externalization of social and environmental costs led to a positive cost–benefit analysis. This was used to justify the project despite stakeholder resistance and prompted decision makers to ignore broader social and environmental impacts and critical stakeholders. Furthermore, a belief that compliance with local law ensures justice may tempt instrumental leaders to assume that their actions are fair. However, law and justice are not the same, especially in weak institutional contexts. In our case, the abuse of the illiteracy and lack of knowledge of indigenous people in land acquisition processes, even if unintentional, constitutes procedural unfairness and injustice. Also, a narrow focus on market efficiency and on business stakeholders can undermine a justice as equity approach, leading to the exclusion of legitimate stakeholders in all phases of the project (see Murphy & Vives, 2013).

#### Responsibility and Sustainability Orientation

Leaders following an instrumental logic have a low degree of accountability for others (Pless et al., 2012). Driven by fiduciary duty, they feel responsible for shareholders only, and consider the consequences of their decisions on other stakeholder groups only if this is in the interest of shareholders (Jones et al., 2007). It is likely that they would continue the shareholder primacy approach, understand the purpose of the firm as profit maximization and pursue its original objective to become the lowest cost producer of aluminium in the world.

However, since it is in the interest of shareholders to avoid lawsuits and negative reputation, the instrumental responsible leader ensures formal *ex-post* responsibility by complying with local laws. In the case, instrumental leaders would argue that the land acquisition complied with local laws and would reject any accountability for the negative impact of the land acquisition on the indigenous population since this is seen as being beyond their control. In addition, economic cost–benefit arguments would be used to prove the legitimacy of the project.

#### Moderation Orientation

According to the RL literature, the instrumental leader is driven by self-interest and neglects the needs and interests of others (Pless et al., 2012; Waldman & Galvin, 2008). As rational and analytical thinkers, instrumental leaders focus on legal side and cost–benefit analysis, often dismissing emotional aspects as “soft” facts. Only in crisis situations and when the efficient functioning and reputation of the firm is at risk would instrumental leaders pay attention to and try to reconcile opposing interests. They would approach them through a formal acknowledgment of other view, but still try to convince others of correctness of own approach by downplaying seriousness, impact, and consequences, and ultimately aim to influence, intimidate and tame “other voices”, instead of balancing and reconciling different views with mutually satisfying outcomes.

Given the fact that the company featured in the case is operating internationally, a global ethics perspective (e.g. Donaldson, 1996) can further enrich the analysis. The instrumental line of reasoning reflects a relativistic approach. It suggests that the company’s leadership will align their actions with the standards of the country in which they operate. When applied in contexts with weak institutions, inadequate regulations and ineffective law enforcement, relativistic decisions will reflect minimal standards only and the consequences can be disastrous for stakeholders and the natural environment (Stahl et al., 2016). It can be concluded that an instrumental logic does not seem to be well suited to helping leaders to avoid doing harm and to do good for a broader group of stakeholders (including those without a voice and future generations).

### Integrative Responsible Leadership: Central Ethical Orientations

#### Humane Orientation

Integrative responsible leaders are driven by humanistic values (e.g., the well-being of others) and virtues that have an intrinsic morality and a strong regard for others (Cameron, 2011; Sison, 2006). This includes a recognition that the stakeholders involved are vulnerable human beings and that stakeholder relations are not only a means, but an end in themselves. In our case, a responsible leader with an integrative logic would not only show concern for the claims of the dominant stakeholders, but also actively care for those without power and urgency (Mitchell et al., 1997). That is, they would have a genuine interest in resolving the conflict, responding to legitimate claims, and alleviate the suffering of indigenous people. Hence, virtuousness in action would mean applying respect, integrity and dignity in interaction with others, including all stakeholders, and demonstrating a proactive and future-oriented engagement to resolve the conflict for the good of all.

#### Justice Orientation

From a utilitarian perspective, integrative responsible leaders, like instrumental leaders, aim to maximize overall welfare through business activities. However, in contrast to the instrumental leader they apply an “act utilitarianism”. This means that certain decisions are based on a utilitarian cost–benefit analysis, while others – specifically those that concern the life and well-being of people – follow a “logic of appropriateness” (March & Olsen, 2011). The latter logic draws on normative-humanistic instead of economic considerations. In the case, it would be seen as inappropriate to destroy the environment and inflict substantial harm on local communities in the region if only a small percentage of local villagers would have the opportunity to profit from the enterprise (e.g., through job opportunities) and/or if the benefits of bauxite resources only last for a decade but negatively affect future generations. In essence, the pursuit of the project would be reevaluated in light of a logic of appropriateness.

#### Responsibility and Sustainability Orientation

Leaders who follow an *integrative* logic are not only guided by conventional morality or moral rules (be these local ones or hypernorms), “but also [by] what the mature person with a ‘good’ moral character would deem appropriate” (Ferrell et al., 2000, p. 54). Maak et al. (2016) argue that leaders following an integrative logic are guided by more than economic thinking and are driven by a social welfare orientation. Integrative leaders consider needs of all legitimate stakeholders, including those termed discretionary but who can neither create urgency nor have power (Mitchell et al., 1997). The social welfare orientation could include a duty to consider the interest of the state to foster economic development, the corporate interest to make profit, and the interest of the affected indigenous communities to have their rights recognized and to lead a dignified life. If the new CEO was to adopt an integrative RL approach, he would review the mission and vision of the firm and develop its strategy towards a balanced triple bottom line approach. This means he would include social and environmental objectives, while maintaining a competitive position in the aluminium market and pursuing a reasonable return for investors.

Furthermore, applying Jonas’ (1984) distinction between formal *ex-post* and substantive *ex-ante* responsibility, it can be postulated that a leader pursuing an integrative logic not only tries to balance different objectives and bottom-lines, but also different norms and standards. Like the instrumental leader, integrators adhere to local laws and norms and ensure formal compliance. However, they also consider broader, universal moral norms and standards – also called “hypernorms” (Donaldson & Dunfee, 1999, p. 52) – and ensure that company practice is in accord with such broadly shared ethical standards (e.g. human rights conventions, international labour laws), which are supported by, for example, prominent non-government organizations (e.g. International Labour Organization), regional government organizations (e.g. OECD, the Organization of American States), global business organizations (e.g. International Chamber of Commerce) and international media, and are in accordance with principles of major religions. This may require rethinking decisions made in the past (such as land acquisition from indigenous people) and considering the implications of actions to be taken in the present and for a sustainable future.

#### Moderation Orientation

A characteristic of integrative leaders is that they integrate rationality and emotions (Pless et al., 2012) and balance opposites (e.g., self-interest and other regard), which can help to regulate excessive self-interest and greed. They understand different viewpoints and consider the interests and emotions of all parties. An integrative leader would aim to find a balance between organizational interests and those of the villagers, with a true interest in generating the best and most sustainable solution for all legitimate stakeholders.

Table 1 provides an overview of the analysis. We conclude this analysis with a reflection on institutional contextual conditions and provide a tentative conclusion.

**Table 1:** Central ethical orientations, RL logics, and most likely decision-making approaches & conflict outcomes

	Characteristics	Most likely decision-making approach / conflict outcome
Humane orientation	Treating others as vulnerable human beings	
Instrumental RL	Driven by materialistic values (extrinsic morality) Others seen as means to an end (e.g. help to create shareholder value) Caring mainly for the needs of business and project financiers	Instrumental interest in conflict resolution to achieve business success and satisfy financiers Interests of indigenous villagers likely to be subjugated to shareholder interests
Integrative RL	Driven by humanistic values (e.g. well-being of others) and virtues (intrinsic morality) Stakeholder relations seen as ends in themselves (strong other regard) Caring for all legitimate stakeholders Applying respect, integrity and dignity in stakeholder interaction	Genuine interest in finding a substantial conflict resolution for the good of all
Justice orientation	Fair and consistent treatment of others	
Instrumental RL	Applying utilitarian economic cost–benefit calculus (CBC) Following rigid utilitarian rules reflecting market efficiency	CBC to justify the project despite stakeholder resistance (myopia of broader social and environmental impacts) Rule rigidity can lead to procedural unfairness and injustice (such as abuse of illiteracy of indigenous people in land acquisition processes)
Integrative RL	Applying “act utilitarianism”: certain decisions are based on a utilitarian cost–benefit analysis, while others follow a “logic of appropriateness”	Re-evaluation of the pursuit of the project in light of a logic of appropriateness
Responsibility and sustainability orientation	Accountability to others, including to future generations, the welfare of society, and the environment	
Instrumental RL	Low degree of accountability for others Fiduciary duty to serve shareholders only Considering consequences of decisions on others only if relevant for financiers/ owners Avoiding lawsuits and negative reputation and ensuring formal ex-post responsibility by complying with local laws	Continued pursuit of organizational vision to become the lowest cost producer of aluminium in the world Defense of land acquisition as it complies with local laws Rejection of accountability for any negative impact of land acquisition on the indigenous population
Integrative RL	High degree of accountability for others Guided by ‘good’ moral character Combining economic thinking with social welfare orientation Considering consequences of decisions on all legitimate stakeholders (including shareholder, government and fringe stakeholders) Complying with local laws and adhering to universal moral norms and standards	Review of the mission and vision of the firm Development of a balanced triple bottom line approach Rethinking decisions made in the past (such as land acquisition from indigenous people) and Considering the implications of actions to be taken in the present and for a sustainable future
Moderation orientation	Temperance and balanced behavior	
Instrumental RL	Focus on cognition, legal side and cost–benefit analysis Urgency of crisis requires temperance and acknowledgement of other perspectives, emotions and suffering	Formal acknowledgment of other views Continued trial to convince others of correctness of own approach Taming “other voices”, instead of balancing and reconciling different views with mutually satisfying outcomes
Integrative RL	Integrating rationality and emotions Balancing of opposites Regulating self-interest Understanding different viewpoints Considering the interests and emotions of all parties	Finding a balance between organizational interests and those of the villagers

While Maak et al. argue that an instrumental RL logic could work “in relatively stable settings with strong institutional arrangements” (2016, p. 463), these conditions are not easily achieved in many parts of the world (Scherer & Voegtlin, 2018). As shown in the analysis above, a weak institutional context can hinder responsible leaders who aim to avoid doing harm and to do good; and an instrumental RL logic fosters a relativistic and morally limited approach. In this sense, we conclude that the instrumental approach is morally inferior.

In regard to the case it can be concluded that an instrumental RL approach based on shareholder advocacy constitutes a minimal approach to responsibility that is not sufficient to respond to the challenges at hand. In fact, the context calls for an integrative RL approach with a proactive leader who acts in the absence of state support as a corporate statesperson (Maak et al., 2016). The latter can be expected to be more effective in dealing with political CSR and stakeholder challenges and to contribute to closing existing governance gaps and generating sustainable outcomes for stakeholders. In the following, we will therefore discuss possible approaches to the situation by applying an integrative RL logic.

## Discussion and Implications: What Could be Done?

The following discussion and application of an integrative RL logic will be guided by the question *“What could be done?”* This question aims to facilitate the generation of broader moral insights and imagination than the normative question “What should be done?” (Zhang et al., 2018).

The discussion is also based on the assumptions that (a) the UAIL project has the potential to positively contribute to regional development, (b) bauxite resources last for several decades, and (c) the firm can provide safe and fair employment for a substantial number of tribal villagers (for a summary see Table 2).

**Table 2:** Discussion overview: What *could* be done

RL approach	Leadership roles	Outcome
Response and responsibility	Communicator	*General:* De-escalating the situation and developing a collaborative solution *Case-specific:* Stop site development process and direct engagement with local stakeholders in a constructive and inclusive dialogue to recognize concerns and needs; together develop a common ground regarding further operations
Responsibility and time	Citizen	*General:* Displaying accountability, transparency, and moral reflection in the course of *ex-post* and *ex-ante* responsibility; acknowledging legitimate rights of local tribes to voice their concerns and be heard *Case-specific:* Re-thinking decisions made in the past; taking action by developing appropriate steps to remedy the harm inflicted on indigenous people (e.g. compensation); shifting the disputed land to nearby barren land; adopting a proactive and inclusive stakeholder engagement approach throughout different project stages to ensure a sustainable future
Responsibility and logic of appropriateness	Servant	*General:* Considering the needs and well-being of all stakeholders affected by the project *Case-specific:* Providing local communities with fair share of profits; investing substantially in infrastructure; hiring and training indigenous people; creating alternative employment opportunities outside the company through vocational training; providing a safe, healthy and fair work environment that adheres to the standards of the ILO
Global consistency and responsible change	Change agent Architect Coach	*General:* Shaping the business and its strategy to reflect transparent and consistent global CSR standards to avoid harm and do good *Case-specific:* Initiating change towards responsible and sustainable business; define a morally advanced company philosophy; adhere to local CSR norms and be consistent with global CSR standards; design and implement a sustainable business strategy and designing a sustainable value chain
Sustainability standards	Steward	*General:* Meeting the highest sustainability standards *Case-specific:* Ensuring safe and clean production processes and minimizing the company’s impact on the environment
Building cross-sector collaboration (CSC)	Boundary spanner	*General:* Partnering with actors from other sectors to develop joint initiatives that resolve some of the pressing social problems related to running the business *Case-specific:* Establishing a partnership with a local social enterprise to ensure sustainable community development; initiating CSC initiatives for providing communities access to health care and insurance
Engagement in multi-stakeholder initiatives	Visionary	*General:* Contributing to sustainable change and sustainable development goals through proactive MSI engagement (e.g. UN Global Compact) *Case-specific:* Advocating for human rights of indigenous people and for sustainable change at the industry and country levels

According to RL theory (Miska et al., 2016; Stahl et al., 2016), a leader of a globally operating firm does not restrict himself or herself to two competing moral imperatives, but applies moral imagination. To recap, moral imagination entails a mental state of heightened awareness of the situation and context of the dilemma, awareness of the role relationship and understanding of other mental models and viewpoints in the moral conflict, and the capacity to challenge dominant operating perspectives, coupled with the ability of moral reflection and the creative ability to envision new perspectives and ways of seeing (Werhane, 1999, 2002). In this sense, the leader will consider the following choices: First, to follow a global approach and ensure universal consistency by applying the same CSR standards (e.g., hypernorms) across subsidiaries in different countries (global CSR integration of standards or short global CSR standardization); second, to apply a locally responsive approach, which implies acknowledging local CSR-related obligations and complying with customs and standards at the local level (local CSR responsiveness); and a new third way, which is to balance both approaches following a logic of appropriateness, or “glocal CSR moderation”. The first choice requires a defined CSR strategy with clear rules of conduct. Such an approach fosters a culture of responsibility and allows the transfer of CSR best practices around the world. However, Stahl et al., (2016, 2018) warn that an unreflective application of standards, rules and policies can lead to cultural arrogance and imperialism. The second option of local responsiveness would ideally lead to a constructive response to local stakeholders, but bears the risk of relativistic approaches, leading to moral blindness and ignorance of broader ethical principles and norms as well as CSR being adjusted down to the lowest standards. The third approach of transnational CSR or “glocal CSR moderation” requires striking an appropriate balance between global consistency and local adaptation in regard to CSR principles and practices. It requires the ability to integrate opposites. Integration of conflicting interests, opposing objectives and differences is a key characteristic of an integrative leader (Maak & Pless, 2006; Pless et al., 2012). The CEO could therefore pursue a “glocal CSR moderation” approach and try to balance local responsiveness and global consistency through his moral compass (based on the central ethical orientations), and by applying moral imagination.

In the following, we will discuss a morally imaginative approach and what this could mean in light of the responsible leadership roles model and a responsible business approach, evident in the application of a logic of appropriateness and global standards, the engagement in cross-sector collaborations and multi-stakeholder initiatives. The responsible leadership roles model classifies the observable behavior of responsible leaders via metaphorical roles (Maak & Pless, 2006) which can be classified as *normative* roles (the leader as citizen, steward, visionary), *relational* roles (servant, weaver/boundary spanner, storyteller/ communicator) and *operational* roles (the leader as change agent, architect and coach).

### Response and Responsibility: The Leader as Communicator

While the company has been responsive to stakeholder concerns at the global level in the past, a combination of weak institutions, inadequate regulations, coupled with a shareholder primacy focus, led to ineffective stakeholder management at the local level. In order to deal with the current conflict a de-escalating approach is necessary to bring different stakeholders together. It requires the leader to take on the roles of *boundary spanner* and *communicator* to initiate a dialogue with *all* legitimate local stakeholders by speaking their language, approaching them openly and with respect and sensitivity, and listening and responding sincerely to their concerns and demands. Inclusion is an important part of RL (Mària & Lozano, 2010). Discourse ethics provides guidance on how organizational leaders can reach consensual agreements with diverse constituencies. Philosophers like Gadamer (2013) and Buber (2010) advocate a humble and respectful dialogue between two or more parties aiming at a healthy consensus. Proponents of Habermasian discourse ethics (Patzer et al., 2018; Voegtlin et al., 2012) highlight conflict mediation, collaborative problem-solving processes and/or creativity and innovation workshops as potential practices to achieve consensual agreements. Proponents of the Bakhtinian approach to discourse ethics (e.g., Trittin & Schoeneborn, 2017) stress the importance of dialogue as a process of stakeholders articulating their positions in a constitutive polyphonic discourse. This occurs regardless of whether a consensual agreement among multiple stakeholders with often conflicting voices can be reached. Within such a polyphonic dialogue,

responsible leaders would encourage participating individuals to bring in various, potentially dissonant voices into the conversation, including the contextual voices of absent stakeholders or wider societal discourses … [and those] that critically address the social and environmental impact of the firm’s business conduct … [and would also] mediate and translate between multiple logics of various organizational and contextual voices. (Trittin & Schoeneborn, 2017, p. 315)

Past experience shows that leaders applying an instrumental logic try to influence and convince stakeholders about the rightness of the organizational approach, while leaders following an integrative approach attempt to develop collaborative solutions (Maak et al., 2016), and facilitate interplay among various stakeholders and their voices (Trittin & Schoeneborn, 2017). The latter could involve stopping the site development process and directly engage with local stakeholders in a constructive dialogue to recognize their views, concerns and needs, and develop common ground regarding further operations.

For instance, BP and its consortium partners faced a similar situation in 2014 when the Rumaila oilfield in southern Iraq (one of the largest oil production sites in the world) faced community protests (see Fig. 4). The leadership decided to stop the work on the new installation until an agreement was reached with the community. They stopped for a period of six months, at a cost of US $100,000 per day, and brought in an external and independent mediator to support the conflict resolution process (BP, 2020). By involving the community and establishing a social contract, BP were able to successfully continue work, while also meaningfully contributing to the community.

### Responsibility and Time: The Leader as Citizen

Coming back to the distinction of *ex-post* and *ex-ante* responsibility, the metaphor of the *citizen* suggests that the leader could show accountability and moral reflection, applying both forms of responsibility. This would entail rethinking decisions made in the past (such as land acquisition from indigenous people) and considering implications of actions to be taken in the present and for a sustainable future. Hence, instead of ‘downgrading’ moral responsibility and exploiting differences of global norms and local standards and traditions, the integrative leader would actively seek to use moral imagination and thus *‘upgrade’ ethical decision-making* by attempting to reconcile global norms and local standards.

#### Ex-post Responsibility

To sustainably resolve the conflict with tribal people and establish sustainable relationships with these legitimate stakeholders, the integrative leader as *citizen* (Maak & Pless, 2006, 2009) could reflect on the underlying causes of the conflict, and acknowledge the legitimate right of local tribes to voice their concerns and be heard. NGOs have argued that the process of land acquisition lacked transparency, fairness and appropriateness in the sense that advantage was taken of the illiteracy of local villagers, and their lack of education and knowledge about market prices. The latter ultimately resulted in human suffering at the local level instead of improved conditions through economic development. According to Murphy and Arenas (2010), lack of transparency and consultations with indigenous communities erodes trust and generates conflict. While land acquisition occurred legally and while he cannot change events in the past, the leader could set up an independent commission to investigate the land acquisition process (for a discussion on justice ethics concerning such situations, see Murphy & Vives, 2013). If the NGOs’ concerns turn out to be justified, the integrative leader could develop appropriate steps to remedy the harm inflicted on indigenous people.

An example of how to approach such a situation can be found in the Australian mining company BHP Billiton (owner of the BHP Tintaya copper mine in Peru) who faced a similar situation around a land acquisition conflict at the beginning of the Millenium (see Fig. 4). The firm was in conflict with local communities in the province of Espinar as well as national and international NGOs. Over a period of three years BHP engaged in a corporate-community negotiation process called Tintaya Dialogue Table, which resulted in 2004 in an agreement of the company to establish a community development fund and compensate villagers for lost land and livelihoods by providing them with land “equivalent to the amont of territory that was expropriated by the state and acquired by BHP Billiton, as well as an additional 25 to 50 percent more land, depending on the quality” (Mego, 2005, p. 1).

Through such an approach the new CEO of UAIL could lay an essential foundation for acknowledging what is important for the villagers, namely a reliable foundation for their existence, and making a substantial contribution to local development as a basis for long-term engagement and the building of public trust. However, in the short-term this would mean that the CEO would have to justify the associated financial investment (following a logic of appropriateness, see also below) as part of the corporate citizenship approach that the company is pursuing.

#### Ex-ante Responsibility

In terms of ex-ante responsibility the new CEO could ensure that the group (1) develops standardized CSR policies and procedures that reflect the highest ethical standards, and (2) adopts a state-of-the-art stakeholder engagement approach that ensures that current and future mining and production processes follow a responsible stakeholder engagement process. A state-of-the-art stakeholder engagement process can be described as being “proactive, collaborative and inclusive … and engage[s] legitimate stakeholders (including fringe stakeholders) in the planning process and in on-going discourse throughout different project stages” based on a jointly accepted approach (Maak et al., 2016, p. 471).

### Responsibility and the Logic of Appropriateness: The Leader as Servant

The integrative responsible leader applies a “logic of appropriateness” (March & Olsen, 2011), which is a form of moral judgement inspired by virtues of reasonableness (Rawls, 1951). Given the considerable financial gains that aluminium production promises (based on the pursued export strategy, rising world market demand, and minimal costs and wages in Odisha), the extraction and production project promises significant returns on investment once it is up and running. The application of a logic of appropriateness and fairness would require reflecting on ways to respond to local needs by giving back to local communities and providing them with a fair share of the profits from resource extraction on the land that they originally cultivated. This could mean reinvesting substantially in infrastructure, access to clean drinking water, medical services, education and job opportunities.

The metaphor of the responsible leader as *servant* implies that the leader puts people and stakeholders first and cares for their well-being and needs. In this spirit, the CEO could ensure that a substantial number of indigenous people are hired and trained by the company to take up jobs and earn a decent salary. He could also get proactively involved in building alternative employment opportunities for other villagers through vocational training. An integrative leader also creates a work environment that protects workers’ health and dignity by ensuring fair, just and inclusive work structures and processes and by providing equal employment and career advancement opportunities for indigenous people as well as women (Pless & Maak, 2004). At the same time they would ensure that local practice adheres to the standards of the International Labour Organization and work towards consistency of standards, closing the gap between global norms and local standards.

### Global Consistency and Responsible Change: The Leader as Change Agent, Architect and Coach

While local responsiveness is an important quality, the integrative leader combines it with global consistency in terms of highest environmental, human rights and responsibility standards. As Miska et al. (2016) showed for Chinese companies, the degree of global CSR standardization often depends on the influence of the state government; if the state influence is greater, the global integration of standards by a MNC will be higher. In a context where no CSR influence is exercised by the state, an integrative leader could fill this void responsibly by initiating positive change and development within the company (Pless & Maak, 2011). Guided by ethical considerations, the leader could act as an ethical *change agent* and *architect* and shape the business and its strategy to reflect transparent and consistent global CSR standards regardless of the context of operations. Moreover, employees at all levels of the organization could be trained and *coached* in regard to enacting the central ethical orientations and applying moral imagination. This could be an important strategic contribution of the new CEO to the development of the company and its multinational parent, which operates on six continents in emerging, developing and developed countries.

### Sustainability Standards: The Leader as Steward

Aluminium production heavily impacts the social and natural environment (Balaton-Chrimes & Haines, 2017; Liu & Müller, 2012). As a *steward* and guardian of values and resources, the integrative leader cares not only about production efficiency, but also about safe production processes (Donaldson, 1996) and minimizing the company’s impact on the environment. The latter is particularly important because tribal people’s lifestyle is often interwoven with the natural environment (e.g., local villagers get their drinking water directly from surrounding open water sources). Guided by the Golden Rule “Do unto others as you would have them do unto you”, the new CEO could place particular emphasis on the highest sustainability standards and the latest technology to minimize the negative impact on the environment and the health and well-being of people. Introducing measurement and monitoring systems, he could ensure regular reporting on SDGs and to the United Nations Global Compact (Rasche et al., 2013). While these seem to be basic standards, such sustainable practice is still far from being the norm in many developing or indeed, in some developed countries.

### Building Cross-Sector Collaboration: The Leader as Boundary Spanner

In our case, the leader is faced with a seemingly insolvable dilemma. While the company can limit its impact on the environment, it cannot avoid adverse impact on the habitat and health of the villagers, who have no access to water and sanitation systems and whose lifestyle directly depends on nature. This situation requires the leader to recognize the situation as a dilemma and to apply compassion and moral insight to resolve it. The ability of “reperceiving” (Shapiro et al., 2006), namely to distance oneself from one’s own internal cognitive and emotional states, and to respond without being fully absorbed by them (Kabat-Zinn, 1990), is discussed as being particularly helpful in such moral conflict situations. It can help leaders to step back from a dilemma, be less reactive, and more open to other choices, thereby supporting more reflective and creative decision-making processes and imaginative moral solutions (Eisenbeiss et al., 2014; Werhane, 2017). A case in point is the German chemical and pharmaceutical company Bayer, which tackled a child labour dilemma in India (see Fig. 4). Instead of staying trapped in a dilemma with the option of either selling their CropScience business or succumbing to the cultural tradition of engaging children in work, they developed a morally imaginative approach:

Bayer created a strategy that was beneficial to the children, the farmers and to its bottom line. It partnered with an Indian NGO that provided remedial schooling for the children of the farmers, children who formerly worked in the fields, so that they would be prepared to enter government schools, and it paid the farmers a premium to use adult labor. The farmers soon found that their efficiency was improved … the children went to school, children’s families did not suffer income loss, and the company gained a valuable product. (Werhane, 2017, p. 216)

Searching for external partners and working in cross-sector collaborations to develop responsible innovative approaches is an important mechanism to approach grand societal challenges and rebuild public trust (Murphy & Arenas, 2010). Integrative leaders driven by a social welfare orientation are likely to see the value of such collaborations and regard extended citizenship engagements of their company as part of the social contract between business and society (Maak et al., 2016). As a *boundary spanner*, the CEO could establish a partnership with a local organization from the non-profit sector to ensure sustainable community development. At the local level this could be a collaboration with a social enterprise like Gram Vikas (GV), which is a globally award-winning organization headquartered in the capital of Odisha. GV helps underprivileged communities in India and Africa to escape the poverty trap and regain dignity by supporting and guiding them to build their own water and sanitation systems (Pless & Appel, 2012). Access to clean drinking water is the access point for a social innovation process that entails democratic community development based on effective and inclusive governance structures that provide equal opportunities for women, Scheduled Castes and Scheduled Tribes. GV contributes to the SDGs by improving health, restoring dignity, empowering women and breaking the vicious circle of poverty (Pless & Appel, 2012). Integrative leaders try to identify, connect and partner with such innovative social enterprises that can help to resolve some of the pressing societal problems interlinked with running the business. Moreover, as a *boundary spanner* the CEO could care for the concerns of people beyond the employment relationship (Davila & Elvira, 2012).

For example, Shell Petroleum Development Company of Nigeria Limited (SPDC) initiated a cross-sector initiative between communities, government and a health care specialist to set up a community health insurance scheme (see Fig. 4). The so called Obio Community Health Insurance Scheme was created as a response to a community request for health care. Instead of shouldering all the responsibility, SPDC created a multi-stakeholder initiative between communities, government and a healthcare specialist which created a win–win-win situation – with poor communities getting access to health care, the regional clinic receiving stable funding, and the oil and gas company benefiting from a better reputation, improved community relations and healthier employees (Luca, 2018). Through such initiatives the new CEO could make a meaningful contribution to local development and build social capital and trust as a corporate citizen, while strengthening the company’s contribution to the SDGs.

### Engagement in Multi-Stakeholder Initiatives: The Leader as Visionary

Maak et al. (2016) point out that integrative responsible leaders are likely to mobilize their organization and provide capabilities to engage in high-involvement multi-stakeholder initiatives due to their social welfare orientation. Such initiatives, especially when combined with a broad membership base and strong participatory elements, can provide leaders “with an institutionalized context to identify, manage and balance diverse stakeholder interests and concerns” (Maak et al., 2016, p. 476). The CEO could consider engaging in a multi-stakeholder initiative (e.g., UN Global Compact) to work and report on the company’s contributions to the SDGs. Such a multi-stakeholder initiative provides a platform to learn from best case examples and to partner with different stakeholders. This is not only a suggested way to achieve the SDGs, but simultaneously provides a network of like-minded companies that enables knowledge exchange for organizational development. Moreover, such engagement could also lead to shared vision formation for sustainable futures. For example, from a virtues perspective (Cameron, 2011; Rego et al., 2012; Sison, 2006) the integrative leader could even take a step forward and act as a *change agent* for sustainability. Based on a sustainable vision, the new CEO could become a spokesperson for clean production and sustainable change in the industry, with the objective of initiating broad change in energy consumption and delivery from electricity (based on coal) towards renewable sources. He could use his power and influence within the company and boundary spanning capabilities within the industry and across sectors to initiate broader change towards sustainability; thereby helping the industry in India to substantially reduce its environmental footprint (for practical guidance see *Guide for responsible corporate engagement in climate policy*: UN Global Compact et al., 2013). Furthermore, he could become an advocate for the protection of the human rights of indigenous people. A means to do this is to use his influence and power to convince the government to sign the Indigenous and Tribal Peoples Convention of the International Labour Organization, also called ILO Convention 169. Similar initiatives were undertaken by BHP Billiton’s leadership in Australia to establish continuing partnerships with Indigenous communities (BHP Billiton, 2001).

This discussion has provided an overview of behavioural approaches that an integrative leader *could* take to generate a sustainable solution to the stakeholder crisis and to build a basis for sustainable stakeholder relationships and public trust in the region and beyond. The approaches discussed range from short-term responses to long-term approaches. Short-term responses are suited to immediately respond to the crisis and to de-escalate the conflict. Medium to long-term initiatives are suited to building sustainable relationships with stakeholders in the state of Odisha and to making substantial contributions to the SDGs.

## Conclusion and Implications for Theory and Practice

In this article, we used the concept of moral imagination to analyse a business case from South Asia as a basis for imagining responsible leadership responses to a dilemmatic crisis situation. This article contributes to RL theory by developing a more fine-grained understanding of cross-culturally shared ethical orientations pertaining to instrumental and integrative RL approaches and their implications. It has also helped to further refine understanding of integrative RL logic, which can be understood as a substantive form of responsibility that involves assuming accountability to a broad range of stakeholders and for consequences of leadership actions and decisions in the past, present and future. It has further extended the understanding of responsibility by introducing a time dimension (*ex-post* and *ex-ante* responsibility). In light of a knowledge gap in research on RL in emerging country MNC, this article has provided new insights for the application of the frameworks of local CSR responsiveness and global CSR standardization. In particular, we argued that an integrative RL approach requires balancing of local and global consideration and the integration of both due to the moderation orientation of integrative RL. Moreover, we also discussed behavioral decisions that this approach entails. Future research should empirically study antecedents and consequences of an integrative RL approach in developing and emerging country MNC. Miska et al. (2016) identify multicultural experience in top management teams as an antecedent for balancing (moderating) local responsiveness and global standardization. However, other factors that help leaders to practise an integrative approach may play an equally important role and need to be studied. At the micro level these factors include moral identity (Eisenbeiss, 2012), the ability to balance contradictions, complex thinking (Maak et al., 2016), and paradoxical leadership (Waldman, 2014). Particularly, the concept of moral imagination should be further explored and empirically studied. At the macro level such factors could include industry and its context, access to responsible business education, and engagement in cross-sector partnerships. Further research should also investigate the impact of country characteristics (e.g., power distance, feminine/masculine society, individualistic/collectivist culture; Hofstede et al., 2010) on leader propensity to apply an integrative RL style.

One approach leaders from emerging and developing country MNC could adopt to foster positive responsible change in their organizations is engaging in multi-stakeholder networks such as the United Nations Global Compact (UNGC). The UNGC is a supranational organization that put CSR on the agenda, fostered and facilitated dialogue on CSR, and worked on establishing “a consensus on global ethical standards for business via its ten general principles” (Voegtlin et al., 2012, p. 179). However, there is a lack of research on whether and how the UNGC delivers on this purpose (Rasche & Waddock, 2014; Williams, 2014). Future research can contribute to research on the effectiveness and legitimacy of the UNGC by empirically tracking over time the CSR and RL changes of new corporate members of the UNGC. Antonakis et al. (2010) recommend various research designs that can be applied to study such causal relationships.

Last, but not least, researchers should investigate the link between relational leadership and integrative RL. The relational ontology of relational leadership as outlined by Cunliffe and Eriksen (2011) can provide further insights into the relational underpinnings of integrative RL and the creation of a respectful and polyphonic discourse, necessary to initiate and practice inclusive stakeholder dialogue.

An important conclusion drawn from the ethical analysis of the paper is that the instrumental RL approach is morally inferior to the integrative RL approach. A number of authors argue that the pressing societal problems (e.g. geopolitical instability, failing states, climate change, social inequality) are too complex to be effectively addressed using the instrumental approach focused on the single firm's advantage (Maak et al., 2016; Stahl et al., 2018). An anonymous reviewer suggested to further examine the relationship between the instrumental RL approach and current societal problems, suggesting that they were caused by utilization of the instrumental RL approach, and arguing that the latter seems to violate one of the basic premises of any sound moral philosophy – and that is the concept of impartiality. This is an important point, in particular since the instrumental RL mindset is still the dominant script in leadership practice and education. In order to gather further evidence and arguments for creating positive change and a paradigm shift towards a new RL understanding, future research should further investigate this link between current societal problems and the role of an instrumental RL mindset. Future research should also further investigate conceptually and empirically the potential of the integrative RL orientation for realizing positive change.

This article has provided substantial insights how challenges of leading business in society can be approached responsibly in an emerging country context. These insights can be particularly useful for business schools, MNC and other organizations for designing responsible leadership development courses and programs to prepare current and future leaders for the challenges of leading businesses successfully and responsibly at local and global levels and for contributing to tackling grand societal challenges. Responsible leaders employ moral imagination and ask what *could* be done to address moral challenges at home and abroad.

## Endnotes

1. These calls have been evolving over decades dating back to 1953 when businessman Howard Bowen first published his book “Social responsibilities of the businessman” (Bowen, 2013). Since then we have seen numerous initiatives in Europe, Asia and the US. Credit needs to be given to the work of numerous change makers, including for example the work of the Caux Roundtable (Young, 2006); the creation of the B-Corps in 2006 (Marquis, 2020); the conscious capitalism movement (Mackey & Sisodia, 2013), and other initiatives of numerous businesspeople, entrepreneurs, and academics in different parts of the world who made a contribution and paved the way for the ideas for a stakeholder perspective, corporate social responsibility, the triple bottom line idea and ultimately responsible leadership.
2. This phenomenon is not restricted to responsible leadership. Behavioral scientists and human evolutionary biologist criticize that studies and claims about human psychology and behaviour draw on samples of a particular segment of the world’s population that Henrich et al. (2010) call Western, Educated, Industrialized, Rich, and Democratic (WEIRD). Results of these studies populate the world’s top journals, and while WEIRD people are a particular group that do not represent the rest of the population, they became the “standard subjects” for studies from which general conclusions are drawn.
3. The male form is used here, because of the fact that the CEO in the case was male

## Appendix

### Methodology

#### Field study: Data gathering

The field study is based in part on interviews with elite informants, and we applied transparency criteria as outlined by Aguinis and Solarino (2019). In light of the complexity of the case, the emergence of events over time and the multitude of stakeholders, we followed a single case study approach (Yin, 2018) with an embedded longitudinal research design. The research setting was complex and difficult to navigate due to the stakeholder crisis. However, one of the researchers was an organizational member of UAIL at the time, and was able to provide important background information. He knew the organizational culture, behavioral norms and power structures, which helped us to get access to the top management team even during the crisis situation and approval to collect data, and helped in the interaction with elite study participants. Data were collected in multiple phases from 2009 to 2012 following both retrospective and observational methods. The retrospective data were collected through interviews after the incidents took place; they also included the company’s archival documents, newspaper clippings, internet information about the company and census data. Observations were made by one of the authors in person through discussions with multiple stakeholders while the incidents happened. Notes were taken to document the observations.

Since the research is field-oriented and not concerned with statistical generalizability, we used non-probabilistic samples, in particular purposive samples which are commonly used in the social sciences (Guest et al., 2006, p. 61). The different stakeholder groups that we studied (top management team members, employees, suppliers, community members, NGOs) were selected sequentially based on their relevance and importance for the research case, “not their representativeness” (Teddlie & Yu, 2007, p. 80).

A combination of sampling techniques was applied. We chose theoretical sampling to study particular viewpoints of the conflict situation in light of stakeholder theory in order to develop a multifaceted picture of the different ethical issues involved in the case. To select participants from specific constituent groups we used snowball sampling. Interviews were conducted with the CEO and the Managing Director of UAIL, with two senior HR managers, 48 employees from different levels and 40 community members. The latter were conducted with the help of a person fluent in the local language. The interviews were semi-structured and conducted for 45 min to one hour, either in person or over the phone. All interviews were transcribed verbatim. Furthermore, we conducted five focus groups, each consisting of 20–25 members of the community, three non-government organization (NGO) representatives and ten employees of two contractors. Focus group interviews were conducted in five project-affected villages. Through each sarpanch (head of a village) we invited participants to the focus group discussions that took place in the panchayat (village council) office. We recorded entire discussions with their due permissions and translated them from the local language to English afterwards with the help of an interpreter. We stopped data gathering once we reached saturation – the point when we did not get any new information through interviews or focus groups (Guest et al., 2006; Krueger & Casey, 2000, p. 26). The data collected were compared constantly to ensure their consistency and accuracy. Validity was established through data triangulation (Yin, 2018).

During the data gathering we also faced some challenges, for instance bringing women into the focus group discussions. Therefore, we collected women’s views individually before the focus group discussions. Another challenge we faced during interactions with villagers was language interpretation. At the initial stage, we worked with simultaneous translation from the local language into the official language. However, we observed a time lag between the villagers’ speaking time and the interpreter’s time needed for translation. To avoid potential data loss, particularly in long discussions, we recorded the entire discussions and translated them afterwards. Due to the sensitivity of the case involving a conflict situation and senior leaders, we did not receive permission to make the raw materials available to other researchers.

## Declarations

### Permission

Permission has been obtained for use of photos in Figs. 1, 2 and Fig. 3; color should be used for these figures in print.
